# Emerging Molecular Targets for Brain Repair after Stroke

**DOI:** 10.1155/2013/473416

**Published:** 2013-01-13

**Authors:** Jerzy Krupinski, Mark Slevin

**Affiliations:** ^1^Cerebrovascular Diseases Unit, Department of Neurology, University Hospital Mutua Terrassa, Terrassa, 08221 Barcelona, Spain; ^2^School of Healthcare Science, Manchester Metropolitan University, Manchester M1 5GD, UK

## Abstract

The field of neuroprotection generated consistent preclinical findings of mechanisms of cell death but these failed to be translated into clinics. The approaches that combine the modulation of the inhibitory environment together with the promotion of intrinsic axonal outgrowth needs further work before combined therapeutic strategies will be transferable to clinic trials. It is likely that only when some answers have been found to these issues will our therapeutic efforts meet our expectations. Stroke is a clinically heterogeneous disease and combinatorial treatments require much greater work in pharmacological and toxicological testing. Advances in genetics and results of the Whole Human Genome Project (HGP) provided new unknown information in relation to stroke. Genetic factors are not the only determinants of responses to some diseases. It was recognized early on that “epigenetic” factors were major players in the aetiology and progression of many diseases like stroke. The major players are microRNAs that represent the best-characterized subclass of noncoding RNAs. Epigenetic mechanisms convert environmental conditions and physiological stresses into long-term changes in gene expression and translation. Epigenetics in stroke are in their infancy but offer great promise for better understanding of stroke pathology and the potential viability of new strategies for its treatment.

## 1. Where Are We?

The classical molecular targets for stroke include those involved in oedema/inflammation control, axonal regeneration/plasticity, neurogenesis/angiogenesis, and events that support recovery. For decades, old targets for stroke were based on observations of molecular and cellular changes after stroke. Numerous inflammatory markers, growth-associated proteins, cell cycle proteins, NMDA receptors, molecules involved in synaptic plasticity, dendritic branching, neural sprouting or extracellular matrix remodelling were key targets. The field of neuroprotection generated consistent preclinical findings of mechanisms of cell death but these failed to be translated into clinical therapies. Many clinical trials were carried out using doses that were already known to be ineffective in preclinical trials, or employing time delays outside the established therapeutic window. Some trials were based on preclinical data showing relatively weak effects or those that were only established in one limited model. Similar problems may occur in the field of neural repair without careful work on the key points associated with clinical translation [1]. The effective delivery of neural repair strategies is another major issue in recovery after stroke. Several growth factors and cytokines have been shown to mediate neurogenesis and angiogenesis [2]. However these are pleiotrophic molecules with likely multiorgan effects when delivered systemically. The fine tuning of approaches that combine the modulation of the inhibitory environment together with the promotion of intrinsic axonal outgrowth needs further experimental work before combined therapeutic strategies will be transferable to clinic trials. It is likely that only when some answers have been found to these issues will our therapeutic efforts meet our expectations [3]. Selective delivery systems, or more selective small molecules, will need to be developed to minimize side-effects in a neural repair therapeutic. Nanomedicine is probably opening new opportunities in this field as it may provide opportunities to deliver larger quantities of drugs with the additional possibility to target therapeutics to specific brain regions (superparamagnetic particles) and deliver to specific cell types following antibody-mediated endocytosis [4].

 Stroke is a clinically heterogeneous disease, with infarcts commonly occurring in different tissue compartments (white matter and gray matter) and brain regions (basal ganglia, cortex, thalamus, brainstem), and occurs most often in aged individuals. Combinatorial treatments require much greater work in pharmacological and toxicological testing. Further, treatments that promote anatomical rewiring will need to be administered in combination with behavioural activity to help “stamp in” patterns of brain rewiring that are adaptive and to avoid the formation of maladaptive patterns of wiring. A promising experimental treatment will need, at the very least, to be tested in several different rodent stroke models and aged animals. Despite these issues, it is becoming clear that the partial recovery that is commonly seen after stroke is associated with a reorganization of brain circuitry, and those methods that can safely and effectively enhance this reorganization could potentially have great clinical value [5]. It is important to remember that all stroke patients exhibit some degree of functional recovery. This process occurs in a matter of days, continues most dramatically for the first month in upper and lower extremity motor function [6] and for up to a year in language and other cognitive modalities [7, 8]. This recovery is only partial, leading to the tremendous long-term personal and financial burdens of this disease [9, 10]. What mediates natural neural repair in stroke and what are the pharmacological targets to promote improved recovery? Many of these processes of structural and physiological change after stroke have been correlated with recovery but the causal mechanisms of neural repair in stroke have not been defined. Axonal sprouting from the cortex contralateral to an infarct into the cervical spinal cord and brainstem ipsilateral to the infarct correlates with recovery of forelimb use [11, 12]. Neurogenesis after stroke is associated with functional recovery, in that blocking mitotic activity after stroke reduces cognitive recovery [13]. The degree of angiogenesis after stroke in humans is correlated with the level of recovery [14]. Stem cell, growth factor, and cytokine therapies that promote functional recovery correlate with increases in angiogenesis and neurogenesis near the infarct [15, 16]. 

 In recent years, the field of neural repair in stroke has identified cellular systems of reorganization and possible new molecular mechanisms. However, conceptual barriers now limit the generation of clinically useful agents. First, it is not clear what the causal mechanisms of neural repair are in stroke. Second, adequate delivery systems for neural repair drugs failed and need to be determined for candidate molecules. Third, ad hoc applications of existing pharmacological agents that enhance attention, mood, or arousal to stroke were unsuccessful. New approaches that specifically harness the molecular systems of learning and memory provide a new avenue for stroke repair drugs. Fourth, combinatorial treatments for neural repair need to be considered for clinical therapies. Finally, neural repair therapies have as a goal altering brain connections, that is, rewiring cognitive maps and active neural networks. These actions may also trigger a unique set of “neural repair side effects” that need to be considered in planning clinical trials [17]. Future research will be needed to address the above limitations in this field and problems in translation from the basic science to poststroke clinics.

## 2. Heading towards the XXII Century

The Whole Human Genome Project (HGP) early in the XXI century ushered in a wave of optimism and anticipation that new therapies and even cures for many diseases would soon be forthcoming [18]. Aside from impressive progress in reducing the costs of genotyping [19], the promise offered by the HGP has been largely unrealized, particularly in relation to stroke [20]. More than 100 Genome-Wide Association studies [21] made possible with the new information provided by the HGP have yielded many interesting findings about the genetics of stroke-related brain injury, but all have generally fallen far short of identifying a genetic basis for vulnerability to cerebral ischemia [22]. With the notable exception of monogenic diseases, genome-wide association studies (GWAS) have generally not been an efficient strategy to elucidate the genetic mechanisms of disease, particularly in complex pathologies such as ischemic cerebral injury [23]. These studies have taught us that most disease pathologies, including those associated with cerebral ischemia, are polygenic and involve highly variable contributions from the genes involved. Such findings raise the important question: why are the genetic components of complex diseases so variable? Polygenomic pathology as well as individual gene variability contributes to stroke as described in many GWAS. Long before the HGP was completed, it was recognized that genetic factors were not the only, or perhaps even not the most important, determinants of responses to some diseases. It was recognized early on that “epigenetic” factors were major players in the aetiology and progression of many diseases [24]. Following completion of the HGP, understanding of epigenetic mechanisms has expanded rapidly, and it is now recognized that epigenetic regulation involves three main categories of mechanisms [25, 26], that is, DNA methylation that attenuates gene expression; enzymes that add and remove acetyl groups to lysine residues in histone proteins and thereby facilitate or inhibit their dissociation for DNA with subsequent increases or decreases in gene expression, respectively; and the pathways that regulate the synthesis and action of micro-RNAs (miRNAs) that regulate mRNA translation. MicroRNAs represent the best-characterized subclass of ncRNAs. Together, these epigenetic mechanisms convert environmental conditions and physiological stresses into long-term changes in gene expression and translation. In contrast to DNA methylation and histone modification, the main function of miRNAs is associated mainly with message translation rather than with gene transcription. The miRNA molecules directly bind mRNA and either retard or accelerate its degradation. In addition, miRNA binding to mRNA can block message translation [27]. The sequences coding for miRNAs often arise from intronic DNA and regulate the gene products coded by adjacent exons. More than 1,000 unique sequences of miRNA have been identified, and together these regulate approximately 30% of all mammalian genes [27]. A single miRNA can help regulate multiple different gene products, and a single gene product can be regulated by multiple different miRNAs. As such, miRNAs play key roles in many cellular functions and are particularly important in cardiovascular biology [28–33]. Expression and action of miRNAs change with development and in response to nutritional stress [34]. The actions of specific miRNA molecules can be inhibited by reverse-sense antagomirs, and these have proven useful in many studies of miRNA function [35, 36].

## 3. The RNA Machine

RNAs are an integral component of chromosomes and contribute to their structural organization [37, 38]. It is now becoming apparent that chromatin architecture and epigenetic memory are regulated by RNA-directed processes where, although the exact mechanisms are yet to be understood, involve the recruitment of histone modifying complexes and DNA methyltransferases to specific loci [39]. Whereas long nonprotein coding RNAs (ncRNAs) have been classically implicated in the regulation of dosage compensation and genomic imprinting in animals [40], they seem to play a much broader role in the epigenetic control of developmental trajectories [39]. For example, ncRNAs may repress gene expression and be associated with complex epigenetic phenomena [41, 42]. Small ncRNAs have been consistently linked with heterochromatin formation via the process of RNA interference (RNAi). Higher-level nuclear organization and chromosome dynamics are also regulated by ncRNAs in a variety of systems. These findings reveal RNA-based mechanistic links between these processes in mitosis. The RNAi pathway along with directed histone modifications also regulates the organization of the nucleolus [43, 44]. In mammals, transcription of long ncRNAs contributes to various processes including T cell receptor recombination [42], maintenance of telomeres [45, 46], X-chromosome pairing required for dosage compensation [47], and inactive X-chromosome perinucleolar localization [48]. The functional organization of chromatin can also be regulated by ncRNAs derived from repetitive elements. Given the abundance of transcribed repetitive sequences, this may represent a genome-wide strategy for the control of chromatin domains that may be conserved throughout eukaryotes. Moreover, such observations and others suggest that a large portion of the genome may in fact be functionally active and that transposon-derived sequences may not be reliable indices of the rate of neutral evolution [49].

## 4. A World of Noncoding RNAs

The examples above provide proof-of-principle that RNA can regulate gene expression at many levels and by using a wide array of mechanisms. The ENCODE project showed that at least 93% of analyzed human genome nucleotides are transcribed in different cells, with similar findings in mice and other eukaryotes, which indicate that there may be a vast reservoir of biologically meaningful RNAs that could greatly exceed the ~1.2% encoding proteins. A fraction of RNAs with short open reading frames (ORFs) potentially encodes peptides but on the other side of the ledger many currently annotated ORFs are not conserved and may be false, which could reduce the number of protein-coding genes in the human genome. There has been debate about whether these ncRNAs are (in the main) functional or simply noise. In some cases, it may be the transcript or merely the act of transcription, or both, that are relevant. Nevertheless, many observations indicate that substantial numbers of ncRNAs are intrinsically functional. These include the fact that many loci produce spliced (and alternatively spliced) transcripts that are developmentally regulated. A large fraction of ncRNAs are expressed in specific regions of the brain, exhibiting precise cellular locations. Some mark new domains within the cell, which means that ncRNAs are also set to have a major impact in cell biology. Comparative analyses indicate that ncRNA promoters are, on average, more conserved than those of protein-coding genes and that ncRNA sequences, secondary structures, and splice site motifs have been subject to purifying selection. Moreover, many ncRNAs are evolving quickly, and some have undergone recent positive selection, as exemplified by HAR1 RNA expressed in the human brain, which contains the sequence conserved in mammals that most rapidly diverged after the human-chimpanzee separation. Although the need for large-scale approaches to explore the function of ncRNAs is evident, a glance at the genome browser will show noncoding expressed sequence tags associated with most genes of interest that may have regulatory functions. ncRNAs are already being identified as markers for cancer and associated with other complex diseases such as coronary disease, diabetes, and Alzheimer's. The elucidation of their function may significantly contribute to the understanding and treatment of such conditions. It may also transform our understanding of the genetic programming of multicellular organisms, particularly as it appears that regulation dominates the information content of complex systems [49]. 

## 5. RNA-Based Epigenetic Mechanisms Implicated in Stroke

The predisposition to and the development of cerebrovascular diseases involves the dynamic interplay between environmental and intrinsic vascular, systemic, and CNS risk factors. Increasing evidence suggests that disruption of these homeostatic and plasticity events involves an array of deregulated epigenetic processes [50]. Appreciation of the potential involvement of epigenetic mechanisms in the incidences and outcomes of stroke has begun to motivate studies of these mechanisms in relation to cerebral ischemia and stroke. DNA methylation has been suggested to contribute to delayed ischemic brain injury in mice and has been correlated with stroke risk in humans. Histone modifications have been implicated in LPS-induced cerebral inflammation and oxidative neuronal injury and may be neuroprotective following ischemia in rodent brains [17]. 

## 6. ncRNAs and RNA Regulatory Networks

The most recently recognized category of epigenetic mechanisms includes the pathways involved in the transcription, processing, and action of a class of short (*≈*20–25 nucleotides) RNA molecules identified as micro-RNAs (miRNAs) [51]. MicroRNAs are first transcribed as longer primary miRNA transcripts that can have multiple functional miRNAs embedded within a single transcript. These primary miRNAs are processed to form mature molecules of approximately 22 nucleotides that regulate the expression of large numbers of target genes through sequence-specific interactions with messenger RNA (mRNA) molecules. MicroRNAs bind to the 3 regulatory regions and to particular coding regions of their cognate mRNAs, leading to sequestration for storage or degradation and to translational repression. Cerebral ischemia in animal models is associated with highly selective and temporally regulated profiles of miRNAs in the postischemic brain [52]. Differential expression of miRNAs in the postischemic brain correlates with differential expression of their target mRNAs, including many implicated in transcriptional regulation, ionic flux, inflammation, and other stress responses. These results suggest that miRNA networks regulate a spectrum of processes in the postischemic brain. MicroRNA-140 is one of the miRNAs that was rapidly up regulated in the brain 3 hours after middle cerebral artery occlusion and sustained for 72 hours. One of the validated target mRNAs for miRNA-140 encodes stromal cell-derived factor 1, which plays an important role in the CNS by mediating neural progenitor cell proliferation and migration and tissue repair after cerebral ischemia [53]. This observation suggests that miRNA-140 may be responsible, in part, for mitigating the regenerative response in the postischemic brain. Furthermore, some miRNAs that are highly differentially expressed in brain tissue can similarly be detected in peripheral blood [54], suggesting not only that these may serve as novel clinical biomarkers but also that these miRNAs may be involved in mediating systemic responses to cerebral ischemia.

Multipotent mesenchymal stromal cells (MSCs) have potential therapeutic benefit for the treatment of neurological diseases and injury. MSCs interact with and alter brain parenchymal cells by direct cell-cell communication and/or by indirect secretion of factors and thereby promote functional recovery. In another study, using Multipotent mesenchymal stromal cells (MSCs) treatment of rats subjected to middle cerebral artery occlusion (MCAo) significantly increased microRNA 133b (miR-133b) level in the ipsilateral hemisphere. In vitro, cultured neurons treated with exosome-enriched fractions from MSCs exposed to MCAo brain extracts significantly increased the neurite branch number and total neurite length. This study provides the first demonstration that MSCs communicate with brain parenchymal cells and may regulate neurite outgrowth by transfer of miR-133b to neural cells via exosomes [55].

Further, microRNAs (miRNAs) regulate formation of myelinating oligodendrocytes. Overexpression of miR-219 and miR-338 as oligodendrocyte-specific miRNAs is sufficient to promote oligodendrocyte differentiation. These findings illustrate that miRNAs are important regulators of oligodendrocyte differentiation, providing new targets for myelin repair [56]. 

**Figure 1:**
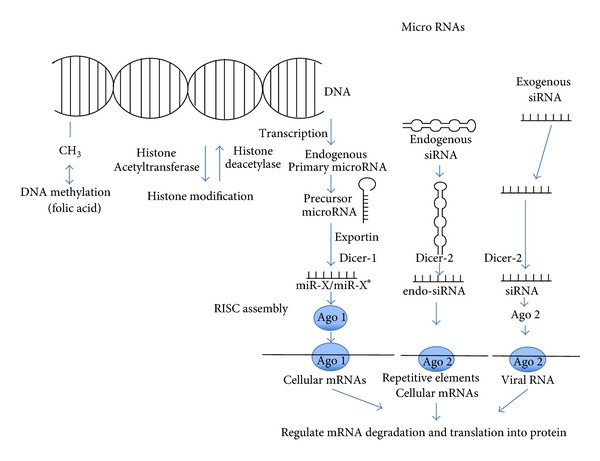
There are three main epigenetic mechanisms. (1) The first includes the mechanisms mediating DNA methylation, typically at cytosine residues in gene promoter regions. These reactions attenuate gene expression and are catalyzed by multiple different isoforms of DNA methyltransferases. An important requirement for these reactions is a methyl donor, typically folic acid supplied through the diet. (2) The second epigenetic category of mechanisms includes the enzymes that acetylate and deacetylate lysine residues on histone proteins. These enzymes regulate chromatin structure and include histone acetyltransferases and histone deacetylases. In general, histone acetylation promotes dissociation from DNA and facilitates gene expression, whereas deacetylation promotes reassociation and reduced gene expression. (3) The third epigenetic category includes the pathways that transcribe, process, and transport microRNA, endogenous short interfering RNAs (siRNAs), and exogenous siRNAs. Endogenous microRNAs are transcribed from nuclear genes into primary microRNA transcripts, which are cleaved into precursor microRNA transcripts. The nuclear protein, Exportin 5, transports precursor microRNAs onto the cytoplasm, where it is cleaved by Dicer to an imperfect miR-X : miR-X* duplex. One strand of the duplex is degraded and the remaining, mature microRNA binds Dicer and Argonaute (Ago) proteins to form RNA-induced silencing complexes (RISCs). MicroRNAs target sequences within cellular mRNAs. Parallel processes in the cytoplasm produce siRNAs derived from endogenous transposons, or from exogenous siRNAs and target cellular or viral mRNAs.

 Long ncRNAs represent another important and emerging subclass of ncRNAs that may also play a role in stroke. Long ncRNAs have roles in local and long-range chromatin remodelling, transcriptional regulation, and alternative splicing and other forms of post transcriptional RNA processing [57]. They are implicated in the development of axonal and dendritic connections and synaptic modulation associated with neural network plasticity. Long ncRNAs may also participate in the generation of the long-term potentiation that underlies learning and memory [58]. An lncRNA can bind to the cyclin D1 gene, a critical mediator of ischemic neuronal cell death [59]. *ANRIL *(NCBI EntrezGene 100048912) is an lncRNA with an unknown function that is associated with the development of atherosclerosis, diabetes, and aneurysms, possibly through effects on vascular smooth-muscle proliferation and migration. *GOMAFU* (NCBI Entrez Gene 440823) is another lncRNA that is expressed in the nucleus of developing neural cells. Although the function of *GOMAFU *is unknown, a case-control association study identified a single nucleotide polymorphism associated with the *GOMAFU* locus as a susceptibility factor for cardiovascular disease [60]. In addition to miRNAs and lncRNAs, other ncRNA transcripts, such as those resembling the virus-like 30 family of interspersed, repeated, mobile genetic elements (i.e., retrotransposons), are also increased in mouse brain after cerebral ischemia. These virus-like 30 ncRNAs are induced by ischemia and paradoxically bound to polyribosomes, although they are not translated. The distribution of these virus 30-like ncRNAs in ribosomal fractions is distinct from the distribution of mRNAs that are translated or translationally repressed and suggests a novel structural or catalytic role for these ncRNAs. Together, these observations imply that the expression and function of several newly identified subclasses of ncRNAs may be associated with the pathogenesis of stroke [61].  Figure 1 represents biogenesis of microRNAs.

## 7. RNA Editing and DNA Recoding in Stroke

This process is intimately linked with ncRNA expression and is another epigenetic process, RNA editing, a mechanism for altering nucleotides in RNA molecules that allows the generation of significant diversity of transcripts in a highly environmental-responsive manner. In transient global cerebral ischemia models, the death of hippocampal CA1 pyramidal neurons is mediated by selective downregulation of an RNA-editing enzyme leading to defective editing of the ionotropic glutamatergic -amino-3-hydroxy-5-methyl-4-isoxazole-propionic acid GluR2 receptor subunit, which influences the vulnerability of hippocampalCA1 pyramidal neurons to ischemia-associated cell death [62]. By alteration of RNA nucleotides, not only does editing have the capacity to change amino acids and modulate splice-site choice in protein-coding transcripts, but it also has roles in ncRNA-related processes such as miRNA localization, target diversification, and function [63]. MicroRNA regulatory network dynamics mediated by RNA editing may be implicated in stroke. For example, miRNA-151 is found in neurons and up-regulated after middle cerebral artery occlusion, and the immature form of miRNA-151 (primary miRNA-151) is subject to RNA editing that influences processing of the primary miRNA into mature miRNA within the CNS [64]. Intriguingly, miRNA-151 is thought to target various cell cycle regulators as well as protein tyrosine kinase 2 (focal adhesion kinase), a nonreceptor tyrosine kinase involved in integrin and growth factor signalling pathways that is differentially regulated after middle cerebral artery occlusion and implicated in modulating neurite outgrowth, neuronal plasticity, and restoration of neural network integrity within the ischemic penumbra [65]. These observations imply that multiple layers of interleaved epigenetic controls that include RNA editing and miRNA regulatory networks are involved in stroke. Another, miR120 is positively correlated with better prognosis in stroke patients and antagonists to miR497, infused prior to stroke, reduce infarct volume. However, to date, no neuroprotective miRNA mimics or antagomirs have been identified that are effective when delivered poststroke. To identify neuroprotective miRNAs, Selvamani et al. studied a known neuroprotectant, Insulin-like Growth Factor (IGF-) 1, for specific miRNA target sites, with the goal of inhibiting these miRNA to elevate local levels of IGF-1 poststroke. IGF-1 is a critical endogenous neuroprotectant and low normal levels of peptide hormone are associated with increased morbidity and mortality in ischemic heart disease and stroke. Exogenous IGF-1 reduces ischemic injury in many species, stimulates stroke induced neurogenesis and promotes neuronal survival, neuronal myelination, and angiogenesis. Two conserved IGF pathway regulatory microRNAs, Let7f and miR1, can be inhibited to mimic and even extend the neuroprotection afforded by IGF-1. Collectively these data support a novel miRNA-based therapeutic strategy for neuroprotection following stroke in experimental model [66]. 

 In addition, the apolipoprotein-B-(ApoB-) editing catalytic subunit (APOBEC) family of RNA editing and DNA recoding enzymes may also play a role in stroke. These enzymes are cytidine deaminases that edit (deoxy)-cytidine to (deoxy)-uridine and act on RNA and DNA molecules [67]. One of the substrates for these enzymes is *APOB *(NCBI Entrez Gene 338) mRNA, which encodes an important apolipoprotein found in chylomicrons and low-density lipoproteins. Mutations of the *APOB *gene and its regulatory region cause dyslipidemias (eg hypobetalipoproteinemia and hypercholesterolemia), and genetic variants of APOBEC1 and APOBEC2 are associated with high levels of serum low-density lipoproteins and increased atherosclerosis [68]. The APOBECs may affect stroke risk through effects on *APOB *mRNA editing; however, APOBECs may play additional roles within the brain. APOBEC-mediated DNA recoding protects the stability of the genome and also enhances its diversity and plasticity [64]. Although these functions have largely been characterized within the immune system, it is intriguing that the APOBEC3 enzyme subfamily has significantly expanded in primates and that certain members (i.e., APOBEC3G) are expressed in postmitotic neurons [69]. Furthermore, accumulating evidence suggests that RNA editing and DNA recoding may be functionally linked through specific classes of reverse transcriptases within the CNS that can mediate RNA-directed DNA modifications [70]. Also, like the immune system, the CNS exhibits exquisite degrees of functional plasticity by modulating cell identity and connectivity. Because of these observations, we have previously suggested that DNA recoding in the brain might represent a novel mechanism for transmitting productive RNA editing events back into the postmitotic neuronal genome [61]. 

This suggests a possible evolutionary mechanism to account for the multigenerational inheritance of complex cognitive and behavioural traits and risk profiles for stroke in response to both productive and adverse environmental events. Apart from the molecular mechanisms responsible for epigenetic regulation, a broad variety of evidence has implicated epigenetic regulation in long-term environmental influences on gene regulation. One of the best-known such examples is the epidemiological work of Pearce, who identified a cohort of Dutch individuals with a uniquely elevated risk of coronary artery disease [17]. The common feature among this cohort was maternal food restriction during the Dutch famine in World War II. These early studies established that foetal nutritional stress could produce life-long changes in the vulnerability to cardiovascular disease, and subsequent work has further established the epigenetic basis of such “vascular programming”. Similarly, other studies have implicated epigenetic mechanisms in long-term responses to hypoxia [71–74] and ischemia [75–77]. Of particular relevance to stroke are findings that miRNA is involved in ischemic preconditioning [79] and may even play a role in ischemic post conditioning. Together, these results emphasize that environmental influences can produce long-term changes in physiological patterns of gene expression through epigenetic mechanisms. 

## 8. Epigenetics and Transient Ischemia

Transient global cerebral ischemia (TGCI) following systemic hypoperfusion is associated with selective and delayed death of hippocampal CA1 pyramidal neurons through the mediation of a series of parallel epigenetic processes. Within vulnerable neurons, there is selective downregulation of ADAR2 and defective Q/R site editing of the ionotropic glutamatergic AMPA, GluR2 receptor subunit, resulting in the expression of the death-promoting calcium permeable GluR2 isoform and associated impairment in GluR2 mRNA and protein expression, receptor assembly, membrane trafficking, and synaptic targeting. Heterogeneity in ADAR2- mediated GluR2 Q/R site editing enhances the vulnerability of hippocampal CA1 pyramidal neurons to global ischemia-associated neurodegeneration. In parallel, TGCI induces the selective expression of REST within these vulnerable neurons with associated suppression of GluR2 and the CA1- selective m-opioid receptor 1 (MOR1) in inhibitory interneurons through a series of histone modifications, including MOR1 promoter H3/4 deacetylation, H3K9 dimethylation and associated recruitment of the G9a histone methyltransferase. This has been postulated to represent a failed attempt of inhibitory interneurons to dampen the excitotoxicity of CA1 pyramidal neurons by disinhibiting GABA release. Ischemia-induced alterations in the histone code may be the result of early dephosphorylation and inactivation of components of the neuronal ERK1 and CREB1 signal transduction pathways that simultaneously reduce expression of the antiapoptotic, bcl2 gene and activate expression of the proapoptotic, caspase-3 effector pathway [80–83]. 

There is also evidence that the more common type of focal stroke syndrome due to occlusion of the middle cerebral artery is associated with aberrant DNA methylation and histone H3 deacetylation, and that systemic administration of a potent HDAC inhibitor reduces the volume of the ischemic infarction whereas concurrent application of an HDAC inhibitor with a DNA demethylating agent confers neuroprotection against mild but not severe ischemic injury [84]. Increasing evidence suggests that intricate epigenetic processes may also operate to modulate premorbid vascular pathology and responses to agents that attenuate ischemic risk factors. For example, a novel deubiquinating enzyme, ubiquitin carboxyl-terminal hydrolase L1 (UCHL1), mutated in a rare inherited form of Parkinson's disease, is normally present in vascular endothelial cells of atherosclerotic lesions of human carotid arteries and attenuates pathological vascular remodeling by inhibiting tumor necrosis factor a-induced NF-kappaB activation [85]. Interestingly, the normal balance of transcriptional activity and associated histone acetylation and methylation that is disrupted in cerebral ischemia depends, in part, on maintenance of the balance of histone H2A and H2B mono-ubiquitylation that is mediated through the actions of UCHL1 [86]. Moreover, statins have recently been shown to act through inhibition of HDAC activity and associated enhancement of histone H3 acetylation [87].

Transient focal ischemia in adult rat brain regulates the expression of microRNAs predicted to target proteins known to mediate inflammation, transcription, neuroprotection, receptor function, and ionic homeostasis in the brain. The mRNA levels for proteins important to microRNA biogenesis pathways, including Drosha, Dicer, the cofactor Pasha, and the precursor microRNA transporter Exportin 5, were not altered after transient ischemia. However, transient ischemia repressed miR-145 expression, which resulted in increased translation of its mRNA target, superoxide dismutase-2, in post-ischemic adult rat brain. It is interesting to note that in silico studies revealed eight microRNAs induced by transient ischemia with complementarity to 877 gene promoters, suggesting that microRNAs also regulate gene expression [88]. There is also specific induction of miR-497 in mouse brain after transient ischemia, and in mouse N2A neuroblastoma (N2A) cells after oxygen-glucose deprivation [86]. Levels of miR-497 correlated with oxygen-glucose deprivation-induced effects on N2A cells: decreased miR-497 suppressed cell death, whereas increased miR-497 increased neuronal loss. As miR-497 directly binds to the 30-UTR of Bcl-2/-w, the knockdown of cerebral miR-497 in mice enhanced Bcl-2/-w protein levels in the ischemic region, attenuated brain infarction, and improved neurological outcome after focal ischemia. These studies show that miR-497 promotes ischemic neuronal death by repressing expression of Bcl-2 and Bcl-w, supporting the role of apoptosis in the pathogenesis of ischemic brain injury [89, 90].

## 9. Molecular Studies of MicroRNAs in Human Stroke

Whole genome expression microarrays can be used to study gene expression in blood, which comes in part from leukocytes, immature platelets, and red blood cells. Since these cells are important in the pathogenesis of stroke, RNA provides an index of these cellular responses to stroke. Human studies show gene expression changes following ischemic stroke. These gene profiles predicted the cause of stroke in 58% of cryptogenic patients. New techniques to measure all coding and noncoding RNAs along with alternatively spliced transcripts will markedly advance molecular studies of human stroke [91].

Platelets are crucial for the maintenance of haemostasis and contribute to thrombosis and vessel occlusion that underlies stroke and acute coronary syndromes. Although platelets are anucleate, they do contain mRNAs and are capable of protein synthesis [89]. Human platelets have been shown to contain microRNAs and Dicer in Ago2 protein complexes, as well as mRNA for the P2Y purinoceptor 12 that is involved in platelet aggregation, suggesting a role for microRNAs in this system [92].

Mutations in mitochondrial DNA are responsible for a spectrum of mitochondrial encephalomyopathies, including mitochondrial encephalopathy with lactic acidosis and stroke like episodes. Although the DNA sequences that harbor these mutations generally do not code for proteins, many of them encode transfer (tRNAs) and ribosomal RNAs (rRNAs). The array of clinical symptoms seen in mitochondrial disorders highlights the functional importance of nonprotein-coding RNAs (ncRNAs) such as tRNAs and rRNAs that are transcribed from nonprotein-coding DNA sequences. In fact, the pathogenesis of a spectrum of neurodevelopmental, neurodegenerative, and neuropsychiatric diseases is increasingly being associated with mutations of ncRNAs [61].

## 10. MicroRNAs as Novel Biomarkers in Brain Ischemia

The brain is a conspicuous consumer of energy resources, and a major consequence of cerebral ischemia is the disruption of energy metabolism and exhaustion of adenosine triphosphate. Because RNA can rapidly be activated, modified, transported, and degraded, it serves as a highly flexible, high fidelity, information encoding, and functional molecule. The ability of RNA molecules to dynamically store, transform, and transmit both “digital” and “analogue” information is a key feature of RNA-based systems [61].

Studies support the potential for microRNAs as novel biomarkers for vascular injury and diseases. Expression profiling of microRNAs in ischemic rat brains revealed significant changes in several micro-RNAs, and some of the microRNAs highly expressed in ischemic brain were detected in blood samples [93]. Peripheral blood examined in ischemic stroke patients revealed differential expression of microRNAs implicated in endothelial cell and vascular function, erythropoiesis, angiogenesis, neural function, and hypoxia, and altered microRNAs were detectable even several months after the onset of stroke [94]. Rat models of ischemia, brain haemorrhage, and kainate-induced seizures also revealed regulated expression of microRNAs in hippocampus and blood in each treatment group, many of which changed >1.5-fold in both tissues [95].

Evidence also suggests that microRNAs serve as effectors in neointimal lesion formation, and in angiogenesis in normal and injured brain. The miR-17–92 cluster is highly expressed in human endothelial cells and miR-92a, a component of this cluster, targets several mRNAs for proangiogenic proteins. Overexpression of miR-92a in endothelial cells blocked angiogenesis, and systemic administration of an miR-92a antagomir led to enhanced blood vessel growth and functional recovery of damaged tissue in mouse models of limb ischemia and myocardial infarction [93]. In a similar vein, profiling of microRNAs in vascular walls after balloon injury revealed that miR-21 is overexpressed in injured vascular tissue, and that miR-21 depletion inhibited formation of neointimal lesions. Depletion of miR-21 decreased cell proliferation and increased cell apoptosis, and targets of miR-21 include the phosphatase and tensin homolog protein (PTEN) and Bcl-2 [96].

## 11. The Era of Epigenomic Medicine

For treatment of stroke, RNA-based therapies and additional epigenetic strategies are extremely promising. Indeed, approaches for gene silencing that use short regulatory ncRNAs, including miRNAs and related short interfering RNAs (RNA interference) have already been used to identify new molecular targets for treating stroke, such as Bcl-2 and 19-kDa interacting protein 3 [97] and carboxyterminalmodulator protein [98]; however, RNA interference-based gene silencing for treating stroke has yet to advance beyond preliminary studies. Therapeutic approaches using other customized oligonucleotides are also being developed for modulation of endogenous RNA transcripts. For example, novel antisense oligonucleotides have now been constructed with the capacity to repair and reprogram aberrant disease-associated RNAs. The mechanism of action of these agents includes alteration of pre mRNA processing (e.g., alternative splicing) and promotion of trans-splicing, which results in the creation of a composite mRNA from 2 separate pre-mRNAs [99]. Although RNA-based approaches such as these are still in their infancy, they offer the potential for dynamic and highly selective reprogramming of gene expression and function. Because of their unique properties, functional RNA molecules may be ideal candidates for a number of future therapeutic strategies. For example, through sequence-specific digital interactions with DNA, RNA-based therapeutic molecules may serve as guideposts for a certain genomic sequence. Through analog interactions with proteins, RNAs may also act as molecular beacons for recruitment of DNA methylation and histone-modifying enzyme complexes to a given genomic locus. Thus, multifunctional RNA molecules with binding domains for DNA and for these enzyme complexes may be used for targeting epigenetic modifications to a single gene locus or to multiple gene loci that harbour a shared genomic sequence. Furthermore, because RNA molecules can interact with DNA, RNAs, proteins, and small molecules, RNA-based therapeutics may also provide the flexibility and specificity necessary to selectively manipulate intricate profiles of gene transcription, posttranscriptional RNA processing, and translation by targeting epigenetic effectors such as nucleosome- and chromatin-remodeling complexes, multiple ncRNAs (e.g., miRNAs and lncRNAs), and RNA editing and DNA recoding enzymes. Although these approaches have yet to be validated, the evolution of CNS drug delivery methods and rapid advances in RNA-based therapeutics, including the advent of RNA aptamers (RNA molecules engineered to bind with high affinity to specific molecular targets such as small molecules, proteins, and nucleic acids), suggest that such strategies are now possible [100]. Future therapies may also be designed to target factors that serve as key modulators of CNS-specific epigenetic events and thereby promote neural cell- and tissue selective epigenetic reprogramming. For example, these strategies may use novel agents that activate or inhibit special AT-rich sequence-binding protein 2 (SATB2), the repressor element-1 silencing transcription factor/neuron restrictive silencing factor (REST/NRSF), and the corepressor for element-1-silencing transcription factor (CoREST). As an environmentally sensitive regulator of neuronal cell fate decisions during development [101], SATB2 modulates neuronal gene expression by promoting coordinate regulation of multiple genes on different chromosomes involved in functionally integrated gene networks. These molecular processes involve dynamic reorganization of the nuclear architecture to allow a seamless link between transcriptional and posttranscriptional processing events and associated RNA quality control mechanisms. SATB2 is also associated with a regulatory lncRNA that is coexpressed with SATB2 [102]. These observations suggest that therapeutic agents targeting SATB2or its associated lncRNA could lead to dynamic reprogramming of neuronal gene expression and even neural cell identityand patterns of neural connectivity that is essential for neural regeneration. REST and CoREST are critical epigenetic factors that mediate predominantly site-specific gene repression, gene activation, and long-term gene silencing for a large spectrum of genes involved in neural development, homeostasis, and plasticity, including but not limited to those that encode growth factors, axon guidance cues, ion channels, neurotransmitter receptors, synaptic vesicle proteins, components of the cytoskeleton, and elements of the extracellular matrix [103].

In addition, REST and CoREST modulate the expression of several classes of ncRNAs, including miRNAs and lncRNAs. These molecules act as dynamic modular platforms for the recruitment of a broad array of epigenetic factors to neural gene loci in which they orchestrate site-specific and genome-wide chromatin remodelling. One of the molecular mechanisms that underlie cell death after transient global ischemia is REST dependent repression of the GluR2 subunit and *μ* opioid receptor 1 [104, 105]. REST also regulates the expression of a significant number of the miRNAs that are differentially expressed after cerebral ischemia [52]. These observations suggest that therapeutic targeting of REST and CoREST may have significant effects on highly integrated epigenetic regulatory mechanisms that could promote reprogramming of neural cells to enhance neural regeneration in stroke by recapitulating developmental events responsible for establishing and remodelling neural cell identity and neural network connectivity. Additional treatment strategies may also be developed to fine-tune epigenetic mechanisms that mediate RNA modifications and trafficking within cells. Among the more salient molecular targets may be regulatory ncRNAs (e.g., miRNAs and lncRNAs), RNA binding proteins, and cytoskeletal proteins (e.g., dyneins and kinesins) that have prominent roles in a diverse array of processes that are under epigenetic regulation, including alternative splicing; editing; nuclear export; stabilization; temporal, spatial, and activity-dependent localization; and translation of RNAs. For example, rationally designed small molecules that bind to miRNAs and modulate their activity are now under early stage development, and these agents may specifically be designed to target miRNAs that are deregulated in stroke [106]. Furthermore, novel therapies may act by selectively influencing the composition of complexes that carry mRNAs, ncRNAs, proteins, and other functionally related factors. These structures, referred to as RNA operons,play key roles in axodendritic transport and mediate local mRNA translation and synaptic plasticity [107]. Higher-order regulatory mechanisms coordinate the dynamics of interrelated RNA operons by modulating their individual components, the kinetics of anterograde and retrograde axodendritic transport and activity-dependent deployment and function of neuronal RNAs. These mechanisms are termed *RNA *regulons. In postischemic neurons, vulnerability to cell death is associated with pathological alterations in RNA operon and regulon dynamics and stress responses that lead to translational [108]. These observations imply that manipulating RNA posttranscriptional processing may be useful in postischemic neurons to promote cellular reprogramming and to selectively activate responses that favorneuronal survival and the maintenance of neural network integrity. Furthermore, RNA operons and regulons are implicated in bidirectional axodendritic transport responsible for relaying RNA editing events from the synapse to the nucleus for DNA recoding within postmitotic neurons. Because these processes are implicated in multigenerational inheritance, therapeutic interventions targeting RNA editing events and associated recoding of the neuronal genome may be implemented to directly alter stroke risk even in future generations. Epigenetic mechanisms are also involved in regulating cell-cell communication, including the active transport of RNAs between adjacent nerve cells through multiple signalling pathways, to more distant sites within the same tissue, to other organ systems through blood-borne routes, and even back to the germline; these processes may represent novel targets for future therapeutic initiatives [109]. Specific transmembrane proteins required for the systemic spread of RNA interference are expressed in the adult brain preferentially in areas associated with learning and memory. Moreover, microvesicles (i.e., exosomes) containing mRNAs and ncRNAs are produced by neural cells and secreted locally and into the peripheralcirculation [110]. These microvesicles may be responsible for cell-cell communication through local and more long distance intercellular RNA transfer because they express cell recognition molecules on their surfaces for selective targeting and uptake into recipient cells, in which mRNAs may be translated and ncRNAs may exert their regulatory effects. Modulation of microvesicle composition and transport pathways may serve as novel targets for regulating anterograde and retrograde signalling across synapses, reinforcing local and long-range neural network connectivity, and signalling to other organ systems (i.e., the immune system) that may play seminal roles in the pathogenesis and evolution of stroke syndromes and associated co-morbidities. As epigenetic processes begin to reveal the many previously hidden layers of functional information embedded within the genome, many future strategies can be envisioned that exploit these processes to develop novel therapies. In fact, the epigenome provides multiple layers of contextual controls that are intricately interlaced and potentially modifiable, and a single epigenetic intervention may even have a cascade of effects on many interrelated processes, including those that may be important for circumventing the pathogenesis and sequelae of stroke. For example, the DNA double helix itself has the potential to form alternative structural conformations with unique epigenetic properties that can be harnessed for the treatment of stroke. Indeed, when the neuroprotective cytokine, colony-stimulating factor 1, is activated by the BRG1 chromatin-remodeling enzyme, a left-handed DNA stereoisomer referred to as a Z-DNA structurecan be found in the region actively being transcribed [111]. The formation of Z-DNA stereoisomers can, in turn, modulate a range of processes responsible for fine-tuning transcriptional events, regulating chromatin architecture, and promoting specific forms of RNA editing [112]. Thus, understanding complex epigenetic mechanisms and their complementary roles in mediating CNS functions, both in health and in disease, is important for developing next-generation technologies to dynamically reprogram neural cells for treatment of complex neurological disease states, including stroke [61]. 

## 12. Final Conclusions

Studies of epigenetic mechanisms in stroke are in their infancy but offer great promise for better understanding of stroke pathology and the potential viability of new strategies for its treatment. Correspondingly, inhibitors of histone modification have been suggested to be neuroprotective in animal models of cerebral ischemia and intracranial haemorrhage. In turn, miRNAs have been shown to play diverse roles in neuronal, glial, and endothelia responses to stroke. In addition, miRNAs have been suggested to regulate the effects of ischemia on aquaporin expression and function and in some cases may be neuroprotective. miRNAs may also help explain gender-based differences in responses to cerebral ischemia. RNA editing, a related epigenetic mechanism that is partly responsible for generating the exquisite degrees of environmental responsiveness and molecular diversity. In addition, the development of future therapeutic strategies for locus-specific and genome-wide regulation of genes and functional gene networks through the modulation of RNA transcription, posttranscriptional RNA processing (e.g., RNA modifications, quality control, intracellular trafficking, and local and long distance intercellular transport), and RNA translation. These novel approaches for neural cell- and tissue-selective reprogramming of epigenetic regulatory mechanisms are likely to promote more effective neuroprotective and neural regenerative responses for safeguarding and even restoring central nervous system function. Data accumulated to date strongly suggest that further studies of these mechanisms are well justified and that future publications resulting from these studies are worthy of careful attention.
